# 

**Published:** 1859-02

**Authors:** 


### LIST OF PAYMENTS.

Drs. James Thweatt, Ga., $3; J. P. Taylor, Ga., $3; J. R. Hood, Ga., $2; J. H. Gibbs, Mississippi, $2; J. Stone, Ga., $3; L. J. Dupree, Ga., $3; James H. Galloway, Miss. $10; John Coston, Ga., $6; J. J. Cowan, Ga., $2; J. R. Biggs, Texas, $4; J. McKey, Ga., $2; T. W. Newsome, Ga., $6, J. F. Alexander, Ga., $9; J. H. Jones, Texas, $9; W. C. Patterson, Ala., $10.

